# Orforglipron, a Small-Molecule Glucagon-like Peptide-1 Receptor Agonist (GLP-1RA), Has Neuroprotective and Anti-Inflammatory Effects

**DOI:** 10.3390/cells15141301

**Published:** 2026-07-21

**Authors:** Yazhou Li, Elliot J. Glotfelty, Pathik Parekh, Buyandelger Batsaikhan, Weiming Luo, Shelley N. Jackson, Brandon K. Harvey, Nigel H. Greig

**Affiliations:** 1 Drug Design & Development Section, Translational Gerontology Branch, Intramural Research Program, National Institute on Aging, National Institutes of Health, Baltimore, MD 21224, USA; pathikhiteshbhai.parekh@nih.gov (P.P.); buka.batsaikhan@nih.gov (B.B.); luowe@grc.nia.nih.gov (W.L.); 2 Cellular Stress and Inflammation Section, Integrative Neuroscience Research Branch, National Institute on Drug Abuse, National Institutes of Health, Baltimore, MD 21224, USA; elliot.glotfelty@nih.gov (E.J.G.); bharvey@intra.nida.nih.gov (B.K.H.); 3 Translational Analytical Core, National Institute on Drug Abuse, National Institutes of Health, Baltimore, MD 21224, USA; shjackson@intra.nida.nih.gov

**Keywords:** glucagon-like peptide-1 receptor (GLP-1R), neurodegeneration, neuroinflammation, neuroprotection, brain insulin resistance

## Abstract

GLP-1 receptor (GLP-1R) agonists, widely used for diabetes and obesity management, have shown preclinical and clinical promise as potential therapeutics for neurological conditions. Until 2026, all U.S. Food and Drug Administration (FDA)-approved GLP-1 drugs were peptide-based, with most requiring daily or weekly subcutaneous injections. Despite their large size and low brain penetrance, several peptide-based GLP-1 drugs are in clinical trials for neurologic indications. Recently, the FDA approved orforglipron as the first small-molecule, non-peptide, orally bioavailable human GLP-1 receptor agonist, and it may offer advantages over peptide-based counterparts. We hence evaluated whether it possesses similar neurotrophic, neuroprotective, and anti-inflammatory attributes. Herein, we utilized human-derived neuronal and microglial cell lines to investigate these properties. Orforglipron activated cAMP signaling and shielded neuronal cells from excitotoxic glutamate and oxidative stress, which are common in neurodegenerative diseases and injuries. Additionally, orforglipron effectively mitigated LPS- and glutamate-induced proinflammatory protein secretion (IL-6, IL-8, and MCP-1) from a human microglial cell line. Actions were replicated under insulin resistance—a potential cause of neurodegenerative conditions—in which orforglipron significantly upregulated phosphorylation of protein kinase B (Akt), providing an additional neuroprotective mechanism. Measured orforglipron brain uptake in rat was low (brain/plasma and CSF/plasma ratios: 0.0078), and in the ballpark of peptide-based GLP-1 drugs. Nevertheless, orforglipron may provide potential value in specific neurological conditions.

## 1. Introduction

The U.S. Food and Drug Administration (FDA) recently approved orforglipron (LY3502970), a first-in-class, orally bioavailable, non-peptide agonist (NPA) of the glucagon-like peptide-1 receptor (GLP-1R) for weight management in overweight and obese adults with at least one weight-related comorbid condition [1]. Unlike traditional peptide GLP-1R agonists that require subcutaneous administration, orforglipron is a small molecule optimized for oral delivery with specificity to the human GLP-1R [2]. Amongst the many peptide-based GLP-1R agonists on the market, only one other formulation (oral semaglutide) exists for oral dosing; however, unlike orforglipron, it requires high relative doses, exhibits low bioavailability, and requires pre- and post-dose fasting and a prescribed quantity of water [3]. Early clinical trials of orforglipron demonstrated substantial reductions in body weight and improved glycemic control, positioning it as a promising next-generation, orally delivered incretin therapy for type 2 diabetes mellitus (T2DM) and obesity [4,5,6,7]. Diabetes patients disproportionately prefer oral daily medications vs. weekly injections (3:1) when initially given an option, with this preference dropping significantly after learning of fasting requirements also usually associated with oral drugs [8]. Since orforglipron does not require fasting or food/liquid restrictions, it may become a desirable alternative for many patients.

Epidemiological and mechanistic evidence increasingly links metabolic dysfunction—especially insulin resistance and T2DM—with heightened risk for neurodegeneration, synaptic loss, and cognitive decline [9]. Diabetes patients who use GLP-1R agonists or dipeptidyl peptidase-4 (DPP-4) inhibitors notably experience a reduced risk for developing dementia [10,11], Parkinson’s disease [12,13], glaucoma [14], and stroke [15,16]. In this context, agents originally developed for metabolic disorders are emerging as promising candidate drugs for repurposing towards neurodegeneration [17,18,19,20,21,22]. Our group and others have demonstrated neuroprotective effects of various peptide GLP-1R agonists in cellular and animal models of neurological disorders [23,24,25,26,27,28,29,30,31], and applications to human neurodegenerative diseases remain promising [22].

The expansive range of preclinical evidence suggests that GLP-1R activation exerts broad neuroprotective actions across multiple experimental models. In preclinical models, GLP-1R agonists reduce microglial and astrocytic activation [32], attenuate neuroinflammation [33,34], suppress oxidative and cellular stress [35,36], support neurogenesis and enhance neuronal survival pathways [37,38], promoting improvement of synaptic plasticity and cognitive performance [39,40,41].

Here we hypothesize that orforglipron shares many of the previously reported neuroprotective attributes observed with peptide GLP-1R agonists. As orforglipron only has affinity to the human GLP-1R, we have undertaken this first evaluation of its neuroprotective effects in strictly human cellular models. Our studies indicate that orforglipron’s utility may extend beyond its approved indications, promoting neurotrophic, neuroprotective, and anti-inflammatory actions in CNS cell culture models. Unlike traditional injectable peptide GLP-1R agonists, orforglipron’s oral bioavailability and small-molecule format may offer advantages in accessibility, dosing flexibility, and potentially CNS penetration. These may provide opportunities for neurodegenerative disease therapy in the future, and hence we evaluated its brain-to-plasma concentration ratio following systemic administration under a semi steady-state condition in rats.

## 2. Materials and Methods

### 2.1. Materials

Orforglipron (LY3502970) was purchased from Selleck Chemicals LLC (Houston, TX, USA). Coelenterazine-H was from Regis Technologies (Morton Grove, IL, USA). Glutamate, dimethyl sulfoxide (DMSO), retinoid acid (RA), RIPA lysis buffer, and other commonly used chemicals and reagents were obtained from Sigma-Aldrich Corporation (St. Louis, MO, USA) unless otherwise indicated. Halt™ Protease and Phosphatase Inhibitor Cocktail (100×) and 4–12% NuPAGE Bis-Tris Gels are from Thermo Fisher Scientific (Waltham, MA, USA).

### 2.2. Cell Cultures

(i) A human GLP-1 receptor (GLP-1R)-overexpressing SH-SY5Y cell line (SH-SY5Y #9 cells), previously characterized by our group [42], was utilized in the present study and was cultured in a 1:1 mixture of Eagle’s Minimum Essential Medium (EMEM; ATCC^®^ 30-2003™, Manassas, VA, USA) and Ham’s F12-K (Kaighn’s) Medium (ATCC^®^ 30-2004™), supplemented with 10% heat-inactivated fetal bovine serum (Gibco™ Cat#10082147, Invitrogen, Carlsbad, CA, USA) and 100 U/mL penicillin-streptomycin (Gibco™ Cat#15140148). Cells were subcultured at a 1:5 ratio every five days using 0.05% trypsin and 0.53 mM EDTA (Invitrogen Cat#AM9912, Invitrogen, Carlsbad, CA, USA). Differentiation: To differentiate SH-SY5Y #9 cells, we first seeded 5 × 10^5^ cells in 35 mm plates in normal culture medium for one day, then changed the medium to differentiation medium containing 1% FBS and 10 µM RA for 5 days (changed the medium once at day 3) [43].
(ii) The human microglial cell line HMC3 (ATCC^®^ CRL-3304™) was obtained from ATCC and cultured in Eagle’s Minimum Essential Medium (EMEM; ATCC, Manassas, VA, USA) supplemented with 10% fetal bovine serum (Gibco™ Cat#10082147) and 100 U/mL penicillin-streptomycin (Gibco™ Cat#15140148). Cells were subcultured at a 1:3 ratio every five days using 0.25% trypsin and 0.53 mM EDTA (Invitrogen Cat#AM9912), and up to 10 passages were used for experiments.
(iii) SH-SY5Y cells stably expressing *Gaussia* luciferase (GLuc) in the endoplasmic reticulum (ER) (SH-SY5Y^GLuc-ASARTDL^) [44] were cultured in DMEM (4.5 g/L D-glucose) containing 2 mM GlutaMAX, 10% heat-inactivated fetal bovine serum (Gibco™ Cat#10082147), and 100 U/mL penicillin-streptomycin (Gibco™ Cat#15140148). These cells were used to broadly assess ER stress caused by ER calcium depletion, a common pathological response across many diseases [45]. GLuc secretion from these cells serves as proxy for a phenomenon known as “exodosis”, the secretion of ER resident proteins following calcium depletion in the ER, and can thus serve as a measure of ER stress [46,47,48]. Up to 15 passages were used for experiments.

### 2.3. cAMP Assays

SH-SY5Y #9 cells were seeded in 24-well plates at a density of 2 × 10^5^ cells/well; the next day, the cells were changed into low-serum (1% serum) media for overnight incubation. On day 3, cells were treated with various concentrations of orforglipron (1, 10, 100 nM) in serum-free media for a period of total 60 min at 37 °C. Cell samples were collected at 0, 5,15, 30, 45, and 60 min after the treatment. First, cells were lysed with 0.1 M HCl containing 0.5% Triton X-100 for 10 min at room temperature. Cell lysates were then collected and centrifuged at 600× *g* at room temperature to remove cell debris. Supernatants were directly used for cAMP measurement. Intracellular cAMP content was determined using the Direct cAMP ELISA kit (Enzo Life Sciences, Inc., Farmingdale, NY, USA), as per the manufacturer’s protocol for the acetylated version.

### 2.4. Cell Membrane Integrity Assays (LDH)

We assessed membrane integrity using the fluorometric CytoTox-ONE™ Homogeneous Membrane Integrity Assay (Promega, Cat. #G7892, Madison, WI, USA), which quantifies lactate dehydrogenase (LDH) released into the culture medium from cells with compromised membranes. LDH release serves as an indicator of cytotoxicity, with higher LDH levels reflecting greater cell damage and reduced viability. The assay was performed in 96-well plates according to the manufacturer’s instructions.

### 2.5. Cell Viability Assays (MTS)

Cell viability was assessed using the CellTiter 96^®^ Aqueous One Solution Cell Proliferation Assay kit (MTS) (Promega, Cat. #G3580), which measures the formazan product that is produced in proportion to viable cell populations (measured using absorbance at 490 nm).

### 2.6. Cellular Reactive Oxygen Species (ROS) Assays

Intracellular ROS levels were quantified using the Fluorescent DCFDA Cellular ROS Detection Assay (Abcam, Cat. #ab113851, Waltham, MA, USA) following the manufacturer’s recommended protocol. This assay employs the fluorogenic dye 2′,7′-dichlorofluorescin diacetate (DCFDA), which diffuses into cells and is oxidized by reactive oxygen species—including hydroxyl and peroxyl radicals—into the highly fluorescent compound 2′,7′-dichlorofluorescein (DCF).

#9 cells were seeded in 96-well plates and serum-starved (0.5% FBS) overnight prior to treatment. Cells were treated with 100 nM orforglipron for 2 h, with or without tert-butyl hydroperoxide (TBHP) at 15 to 75 μM. These TBHP concentrations were selected based on preliminary experiments showing robust ROS induction in #9 cells. Fluorescence intensity corresponding to DCF formation was measured using a plate reader with excitation at 495 nm and emission at 529 nm.

### 2.7. Gaussia Luciferase Secretion Assay

Previous work indicates that exodosis is a feature of glutamate excitotoxicity [46]. Thus, we employed the SH-SY5Y^GLuc-ASARTDL^ cells to monitor GLuc secretion following a glutamate challenge. GLuc was quantified from culture supernatants collected at multiple time points from the same 96-well plate. A 5 μL aliquot of medium was transferred to an opaque-walled plate for each measurement. The GLuc substrate consisted of PBS supplemented with 5 mM NaCl and 10 μM coelenterazine (Regis Technologies, Morton Grove, IL, USA). Coelenterazine stock (20 mM) was prepared in acidified methanol (10 μL of 10 N HCl/mL methanol) and stored at −80 °C as single-use aliquots. The substrate was equilibrated to room temperature for 30 min before use. Luciferase activity was detected using a Biotek Synergy II plate reader (BioTek Instruments, Inc., Winooski, VT, USA) equipped with an injector system, which dispensed 100 μL of substrate into each well for immediate luminescence measurement. Vehicle controls were included at concentrations equivalent to treatment conditions.

### 2.8. Proinflammatory Cytokine Measurement (ELISAs)

After treatment, cell media was collected for use in cytokine enzyme-linked immunosorbent assays (ELISAs). Human IL-6, IL-8 and MCP-1 levels in cell culture media samples were measured utilizing BioLegend’s corresponding ELISA MAX™ Deluxe Sets (430516, 431504, and 438804, San Diego, CA, USA) in accordance with the manufacturer’s protocol. Assay plates were read using an Infinite m200 Pro plate reader with i-Control software Version 2.0.10.0 (Tecan, Männedorf, Switzerland).

### 2.9. Western Blot Analysis

After differentiation with RA, SH-SY5Y #9 cells were first cultured for 24 h in either normal culture media with 1% FBS or high-glucose (30 mM), high-insulin (100 nM) culture media with 1% FBS to induce an insulin-resistant state. Thereafter, SH-SY5Y #9 cells were challenged with fresh media containing 100 nM insulin w/o 100 nM orforglipron for 1 h. Cells were then harvested in ice-cold RIPA lysis buffer (Sigma, St. Louis, MO, USA) with protease and phosphatase inhibitors. The lysates were subsequently collected and centrifuged at 10,000 rpm for 10 min at 4 °C. Next, the protein concentrations in the supernatant fluid of the lysates were determined using Pierce BCA protein assay reagent (Thermo Scientific, Rockford, IL, USA). Protein (40 µg) was separated by 4–12% NuPAGE Bis-Tris Gels and then transferred onto a methanol-activated Immobilon^®^-FL PVDF Membrane (Millipore, Burlington, MA, USA). Membranes were blocked with Intercept^®^ Blocking Buffer (TBS) for 1 h, were washed with TBST, and were then incubated in primary antibody overnight at 4 °C followed by secondary antibody incubation (IRDye 680RD or IRDye 800CW) for 1 h at room temperature in the dark. After final washes and a rinse with TBS to remove Tween, each membrane was scanned, and bands were quantified on a LI-COR Odyssey imager. Antibodies used for Western blot and their dilutions were (1) Akt Antibody #9272, (Cell Signaling, Danvers, MA, USA) (1:1000); (2) Phospho-Akt (Ser473) (D9E) XP^®^ Rabbit mAb #4060 (Cell Signaling) (1:2000); (3) GAPDH Loading Control Monoclonal Antibody (1:5000) (Invitrogen).

### 2.10. Pharmacokinetics (In Vivo Study)

Animals: Eight 8-week-old Fischer 344 rats (4 males, 4 females) (Charles River Laboratories, Wilmington, MA, USA) were used in this study (approved National Institute on Aging, Intramural Research Program, NIH, Animal Care and Use Committee animal protocol 331-TGB-2027). Rats weighed 101–130 g at the time of the experiment and were maintained at 25 °C in a 12/12 h light/dark cycle with access to food and water ad libitum. Orforglipron was administered by the intraperitoneal (i.p.) route at a dose of 3.6 mg/kg of body weight. After 4 h, the rats were euthanized, and blood, CSF, and brain were collected for drug concentration analysis. Blood was immediately centrifuged (10,000× *g*, 60 s, 4 °C), and plasma was removed and immediately frozen (−80 °C). CSF was directly frozen at −80 °C. The brain was rapidly excised, and the right cerebral hemisphere removed and immediately frozen (−80 °C).

### 2.11. Tissue Sample Preparation and Orforglipron Quantification

Drug standards: Stock solutions of orforglipron were diluted in DMSO and used to spike blank matrix at calibration standards of 0.5, 1, 2.5. 5, 10, 25, 50, 100, 250, 500, 1000 ng/mL.

Plasma sample preparation: Extraction was performed by protein precipitation with 150 µL of acetonitrile in 0.1% formic acid added to 50 µL plasma in a 1.5 mL sample tube. The samples were then vortexed (10 s), shaken (4 °C, 5 min at 1000 rpm) with a ThermoMixer C (Eppendorf, Enfield, CT, USA), and then centrifuged (10,000× *g*, 10 min, 4 °C). Next, 100 µL supernatant was transferred to a LC sample plate and transferred to the autosampler, where 10 µL was injected into an Ultra-High-Performance Liquid Chromatography–Mass Spectrometry (UHPLC-MS) system.

CSF sample preparation: Drug extraction from CSF samples (15 µL) followed the protocol for plasma (with proportional reductions in acetonitrile in 0.1% formic acid (45 µL) to ultimately generate 40 µL of supernatant, of which 10 µL was injected into a UHPLC-MS system).

Brain sample preparation: Rat brains were weighed and placed into a 7 mL homogenizing tube with ceramic beads. Ultrapure water was added at 4 mL per g of tissue, and the tissue was homogenized (Bead Ruptor Elite homogenizer, Omni International, Kennesaw, GA, USA). Extraction was performed by protein precipitation with 300 µL of acetonitrile in 0.1% formic acid added to 100 µL tissue homogenate in a 1.5 mL sample tube. The samples were then vortexed (10 s), shaken (4 °C, 5 min at 1000 rpm) with a ThermoMixer C (Eppendorf, Enfield, CT, USA), and then centrifuged (10,000× *g*, 10 min, 4 °C). Next, 200 µL supernatant was transferred to a 0.5 mL sample tube and centrifuged (10,000× *g*, 10 min, 4 °C). Finally, 100 µL supernatant was collected and pipetted into a LC sample plate and transferred to the autosampler, where 10 µL was injected into a UHPLC-MS system.

UPHLC-MS: Sample analysis was performed using a Vanquish UHPLC system (ThermoFisher, Waltham, MA, USA) with tandem Orbitrap Exploris 120 mass spectrometer (ThermoFisher, Waltham, MA, USA). Reverse phase chromatography was performed using an Accucore Biphenyl 2.1 × 50 mm, 2.6 µm column (Thermo Fisher Scientific, Waltham, MA, USA) with 10 mM ammonium formate in 0.1% formic acid as mobile phase A and ACN in 0.1% formic acid as mobile phase B. The flow rate was 0.5 mL/min and the solvent gradient for mobile phase B was as follows: 0 to 1.0 min held at 25%, 1.0 to 5.0 min increased from 5% to 95%, from 5.0 to 7.0 min held at 95%, 7.0 to 8.0 min decreased from 95% to 25%, and then held at 25% to 9.0 min. Analysis was performed in positive ion mode with a full scan mass range of 850–950 *m*/*z* and mass resolution mode of 60K. Ionization was conducted using a heated electron spray ionization (HESI) source. Xcalibur (Xcalibur Version 4.7.69.37, ThermoFisher, Waltham, MA, USA) software was used to integrate and report peak area for the M+H ions for orforglipron (883.3850 *m*/*z* at a retention time of 4.78 min). Data was plotted and fitted to a standard curve to interpolate unknown values. The analytical range of the assay was as follows: plasma 0.57–1132 pmol/mL, CSF 0.57–113.3 pmol/mL, and brain 2.00–1133 pmol/g.

### 2.12. Statistical Analysis

All data are presented as mean ± SEM values throughout, with the number of observations (N) detailed within respective Figure legends. GraphPad Prism software Version 10.1.2 was used for statistical analyses: one-way ANOVA was used with post hoc Dunnett’s or Tukey’s multiple comparisons tests.

## 3. Results

### 3.1. Orforglipron Time- and Dose-Dependently Increases Intracellular cAMP Levels in Neuronal Cells

**Figure 1:**
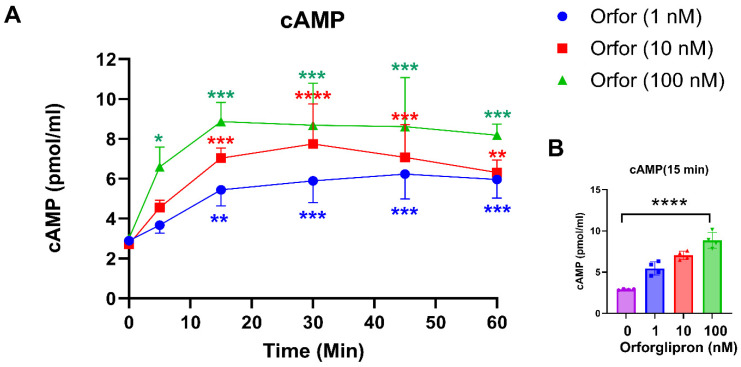
Orforglipron induces a time- and dose-dependent increase in intracellular cAMP levels in SH-SY5Y #9 cells. Cells were treated with increasing concentrations of orforglipron (1, 10, or 100 nM), and time-dependent (0, 5, 15, 30, 45, 60 min) intracellular cAMP levels were quantified using a cAMP ELISA assay. Purple: controls; Blue: orforglipron at 1 nM; Red: orforglipron at 10 nM; Green: orforglipron at 100 nM. Data are presented as mean ± SEM (n = 4). One-way ANOVA was used for statistical analysis. (**A**) Post hoc Dunnett’s multiple comparisons test * *p* < 0.05; ** *p* < 0.01; *** *p* < 0.001; **** *p* < 0.0001 when compared to time zero. (**B**) cAMP levels at 15 min, post hoc Tukey’s multiple comparisons test, **** *p* < 0.0001 (Summary). * *p* < 0.05 any two groups comparison.

Treatment of SH-SY5Y #9 cells with orforglipron produced a time- and dose-dependent increase in intracellular cAMP levels (Figure 1A). At all evaluated concentrations (1, 10, and 100 nM), cAMP levels rose rapidly and reached peak values within the first 10–20 min, after which cAMP levels remained stable for the course of the experiments, likely due to orforglipron’s long half-life (25–68 h) [49]. At 100 nM, cAMP levels peaked at approximately 8.9 pmol/mL at 15 min, significantly higher than those observed at 10 nM and 1 nM (≈7 pmol/mL and ≈5.4 pmol/mL, respectively; *p* < 0.05) (Figure 1B). These findings demonstrate that orforglipron elicits a concentration-dependent activation of adenylyl cyclase and accumulation of intracellular cAMP, consistent with engagement of a Gs-coupled receptor signaling pathway.

### 3.2. Orforglipron Protects Membrane Integrity from Glutamate-Induced Damage in Neuronal Cells

**Figure 2:**
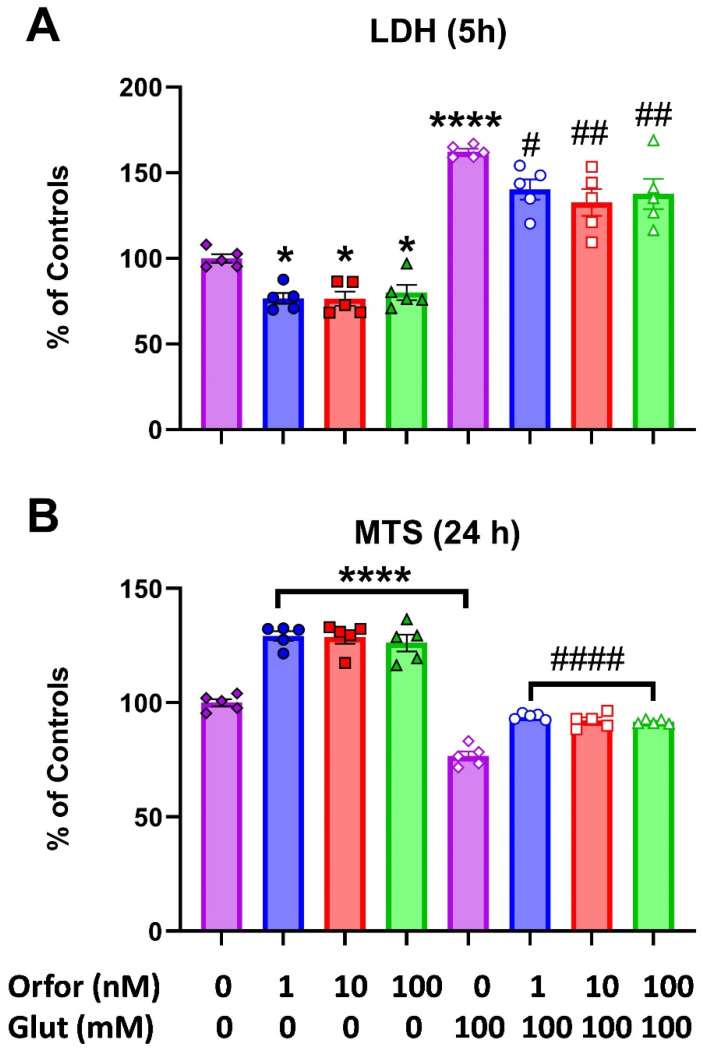
Orforglipron protects membrane integrity and increases cell viabilities in neuronal cells. (**A**) Neuronal cells were pretreated with orforglipron (1–100 nM) for 30 min before exposure to glutamate (100 mM) for 5 h. Purple: controls; Blue: orforglipron at 1 nM; Red: orforglipron at 10 nM; Green: orforglipron at 100 nM. Cell membrane integrity was assessed by measuring LDH released into the culture medium and expressed as a percentage of the untreated control. Glutamate markedly increased LDH release, indicating membrane damage, whereas orforglipron co-treatment dose-dependently reduced this effect. Bars represent mean ± SEM (n = 5). Two-way ANOVA was used for statistical analysis. Post hoc Dunnett’s multiple comparisons test shows * *p* < 0.05 and **** *p* < 0.0001 versus control group; # *p* < 0.05 and ## *p* < 0.01 versus glutamate alone. (**B**) Orforglipron enhances neuronal viability in neuronal cells exposed to glutamate. SH-SY5Y #9 cells were treated with increasing concentrations of orforglipron (1–100 nM) for 24 h, either alone or in combination with glutamate (100 mM). Cell viability was assessed by MTS assay and expressed as a percentage of untreated control. Orforglipron alone modestly increased cell viability, indicative of neurotrophic activity, whereas co-treatment with orforglipron significantly mitigated glutamate-induced decreases in viability across all concentrations. Data are shown as mean ± SEM (n = 5). Two-way ANOVA was used for statistical analysis. **** *p* < 0.0001 versus control group; #### *p* < 0.0001 versus glutamate-treated group.

Orforglipron (1–100 nM) treatment alone slightly reduced LDH at the basal levels (post hoc Dunnett’s multiple comparisons test, ^&&^ *p* < 0.01 versus control group). However, orforglipron protected neuronal cells from glutamate-induced membrane damage, as reflected by significantly reduced LDH release. SH-SY5Y #9 cells exposed to glutamate (100 mM, 5 h) resulted in a significant elevation in extracellular LDH levels to ~162% of control values (*p* < 0.0001), indicating a substantial loss of membrane integrity (Figure 2A). Co-treatment with orforglipron markedly attenuated glutamate-induced LDH release in a concentration-dependent manner. At 10 nM and 100 nM, orforglipron reduced LDH levels to approximately 132% and 137% of control values, respectively (*p* < 0.01 and 0.05 vs. glutamate alone), whereas no significant decline in LDH was evident at 1 nM (Figure 2A). These results suggest that orforglipron confers acute protection against excitotoxic injury by preserving membrane integrity under glutamatergic stress.

### 3.3. Orforglipron Is Neurotrophic and Protects Cell Viabilities Reduced by Glutamate in Neuronal Cells

Orforglipron exhibited neurotrophic effects under basal conditions and enhanced neuronal survival following glutamate-induced toxicity in SH-SY5Y #9 cells. Treatment with orforglipron alone (1–100 nM) for 24 h increased absorbance in MTS assays to approximately 125% of control values across all concentrations (*p* < 0.0001) (Figure 2B), indicating a modest but consistent neurotrophic effect. Exposure to glutamate (100 mM) markedly reduced cell viability to approximately 76% of controls (*p* < 0.0001). Co-treatment with orforglipron attenuated the glutamate-induced reduction across all doses of orforglipron, restoring cell viability to over 90% of control values (*p* < 0.01 vs. glutamate alone) (Figure 2B). These findings demonstrate that orforglipron not only promotes neuronal cell growth under basal conditions but also significantly protects against glutamate-induced loss of viability.

### 3.4. Orforglipron Ameliorates Oxidative Stress in Neuronal Cells

**Figure 3:**
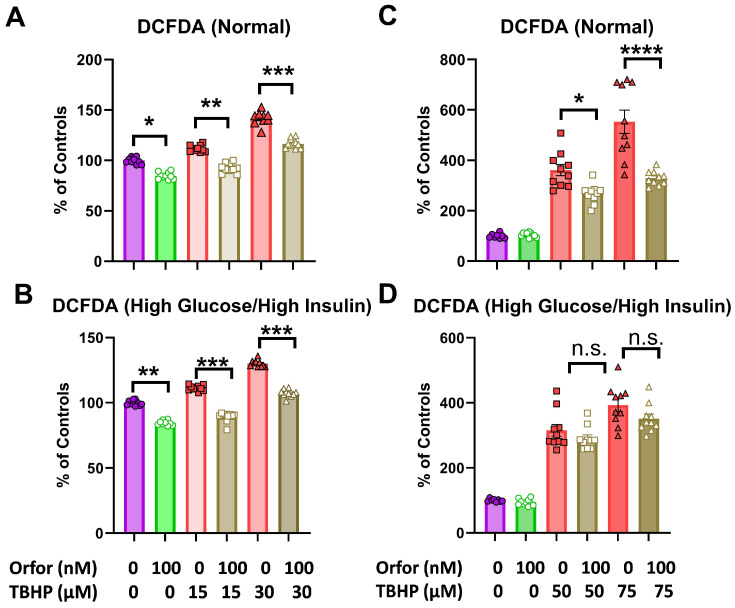
Orforglipron ameliorates oxidative stress in neuronal cells under both normal and insulin-resistant conditions. SH-SY5Y #9 cells were treated with orforglipron (100 nM) for 2 h, either alone or in combination with TBHP, at both low doses, 15–30 uM (**A**,**B**), and high doses, 50–75 µM (**C**,**D**), which induced oxidative stress under normal (**A**,**C**) or insulin-resistant conditions (**B**,**D**). Intracellular ROS levels were assessed using a fluorescent DCFDA Cellular ROS Detection Assay and expressed as a percentage of untreated control. Data are shown as mean ± SEM (n = 5). One-way ANOVA was used for statistical analysis. Post hoc Tukey’s multiple comparisons test shows significance of orforglipron effects at different concentrations of TBHP, * *p* < 0.05; ** *p* < 0.01; *** *p* < 0.001; **** *p* < 0.0001; n.s.: no significant difference.

Orforglipron ameliorates oxidative stress in neuronal cells under normal or insulin-resistant conditions. An insulin-resistant condition was induced by culturing cells in high glucose (30 mM) and high insulin (100 nM) for 24 h. As illustrated in Figure 3, neuronal cells exposed to Tert-Butyl Hydrogen Peroxide (TBHP: a stable oxidative stress-inducing hydrogen peroxide) at concentrations between 15–75 µM exhibited an elevation in ROS levels in a dose-dependent manner, reaching 553% of the ROS level in controls, as detected by a fluorescence-based ROS assay. Under both normal and insulin-resistant culture conditions, treatment with orforglipron alone at 100 nM mildly reduced basal ROS levels compared to the vehicle control in SH-SY5Y #9 cells. Whereas TBHP dose-dependently increased intracellular ROS levels, co-administration of 100 nM orforglipron resulted in a clear reduction in ROS accumulation across all TBHP doses under normal culture conditions (at the highest TBHP concentration of 75 µM, orforglipron reduced ROS levels by 41%) (Figure 3A,C). Under the insulin-resistant culture condition, however, a decline in ROS accumulation was only observed at lower TBHP doses (15 or 30 µM) but not higher ones (50 or 75 µM) when ROS levels reached more than 3-fold the control level (Figure 3B,D). This reveals that the anti-oxidative stress effect of orforglipron is partially preserved under these conditions. These findings demonstrate that whereas orforglipron effectively mitigates oxidative stress at lower levels in neuronal cells in both normal and insulin-resistant conditions, this ability is compromised at higher oxidative stress levels under insulin-resistant culture conditions.

### 3.5. Orforglipron Dose-Dependently Ameliorates Glutamate-Induced ER Stress in Neuronal Cells

**Figure 4:**
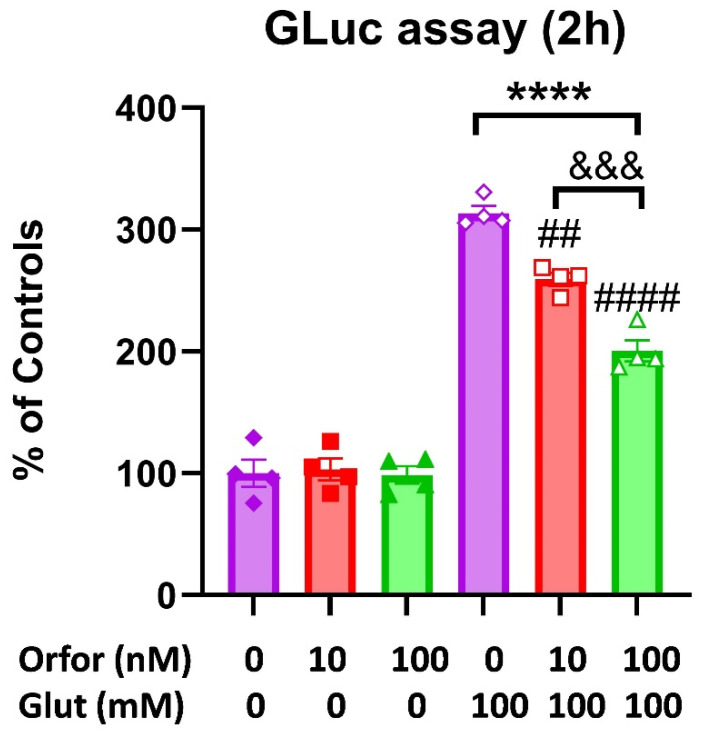
Orforglipron dose-dependently ameliorates glutamate-induced ER stress in neuronal cells. SH-SY5YGLuc-ASARTDL cells were treated with orforglipron (10 or 100 nM) for 2 h, either alone or in combination with glutamate (100 mM). ER stress was assessed using the GLuc assay and expressed as a percentage of untreated control. Data are shown as mean ± SEM (n = 3). One-way ANOVA was used for statistical analysis. Post hoc Tukey’s multiple comparisons test shows **** *p* < 0.0001 versus control group; ## *p* < 0.01, #### *p* < 0.0001 versus glutamate-only group; &&& *p* < 0.001 between treated groups showing a dose-dependent effect.

As evident in Figure 4, exposure of neuronal cells to 100 mM glutamate for 2 h induced a marked increase in ER stress, as measured by GLuc secretion from the SY5Y^GLuc-ASARTDL^ cells (rising to ~313% of control levels). Orforglipron alone at 10 nM or 100 nM had no significant effect on baseline ER stress levels. However, combining orforglipron with glutamate challenge dose-dependently attenuated the glutamate-induced ER stress response: 10 nM orforglipron reduced the signal to ~259% of control levels, whereas 100 nM orforglipron further lowered it to ~200% of the control value (achieving a 1/3 reduction) (Figure 4). These data indicate that orforglipron provides protective effects against glutamate-induced ER stress in a concentration-dependent manner, without altering the ER stress response under basal conditions.

### 3.6. Orforglipron Reduces Pro-Inflammatory Cytokine Levels in Microglial Cells

**Figure 5:**
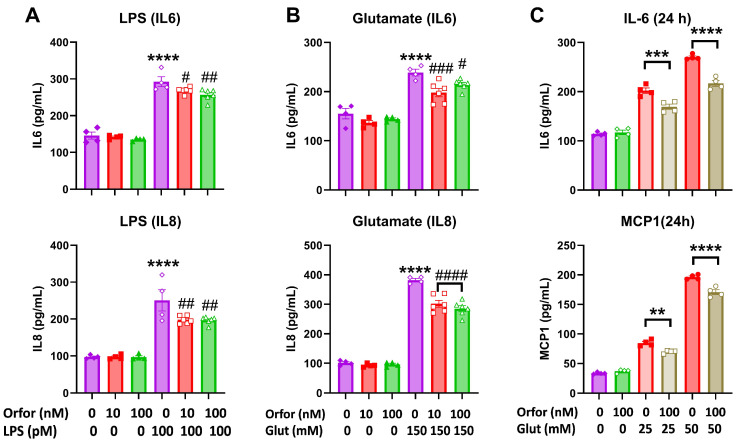
Orforglipron reduces proinflammatory cytokine levels in human microglial cells (HMC3). HMC3 cells were challenged with (**A**) LPS (100 ng/mL) or (**B**) a high concentration of glutamate (150 mM) w/o the presence of orforglipron (10 or 100 nM) for 24 h. Or (**C**) HMC3 cells were exposed to low concentrations of glutamate (25 or 50 mM) in the presence of 100 nM orforglipron for 24 h. Proinflammatory protein (IL-6, IL-8, and MCP-1) levels were then quantified in culture media by ELISA (n = 4–6). One-way ANOVA was used for statistical analysis. Post hoc Tukey’s multiple comparisons test shows **** *p* < 0.0001 versus control group; # *p* < 0.05, ## *p* < 0.01, ### *p* < 0.001, #### *p* < 0.0001 versus LPS or glutamate-only group; ** *p* < 0.01, *** *p* < 0.001, **** *p* < 0.0001 between groups.

As shown in Figure 5, stimulation of human microglial cells (HMC3) with LPS (100 ng/mL) (Figure 5A) or a high concentration of glutamate (150 mM) (Figure 5B) for 24 h significantly upregulated the release of pro-inflammatory cytokines, including IL-6 and IL-8, as measured in culture media by ELISA. Whereas treatment with orforglipron alone (10 nM or 100 nM) did not alter basal cytokine levels secreted by unchallenged microglia, co-administration of orforglipron with LPS or glutamate challenge resulted in a suppression in both IL-6 and IL-8 production. In Figure 5C, exposure to low concentrations of glutamate (25 and 50 mM) dose-dependently increased the expression of the pro-inflammatory cytokines IL-6 and MCP-1 in microglial HMC3 cells, as compared with the untreated control group. Treatment with 100 nM orforglipron significantly attenuated this glutamate-induced elevation of both IL-6 and MCP-1 levels. A similar response on cytokine reductions by orforglipron was obtained with 48 h glutamate exposure (Supplementary Materials). These findings demonstrate that orforglipron suppresses both LPS and glutamate-induced pro-inflammatory signaling in microglial cells, denoting a potential anti-inflammatory and neuroprotective action.

### 3.7. Orforglipron Augments pAkt Signaling Under Both Normal and Insulin-Resistant Conditions in Neuronal Cells

**Figure 6:**
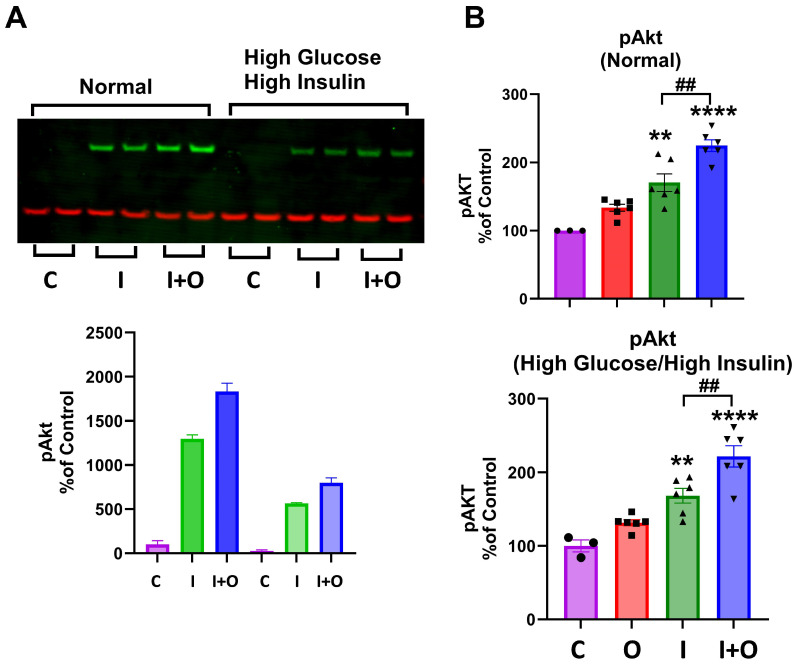
Orforglipron enhances insulin-induced pAkt levels in both normal and insulin resistance conditions. SH-SY5Y #9 cells were first differentiated and then cultured under both normal and high-glucose, high-insulin conditions for 24 h. Cells were treated with 100 nM insulin w/o 100 nM orforglipron for 1 h. pAkt protein levels were quantified by Western blot. (**A**) Representative Western blot with quantification of protein density; (**B**) Quantitative analysis of multiple Western blot results (n = 3). C is control; O is orforglipron alone; I is insulin alone; I + O is insulin plus orforglipron. One-way ANOVA was used for statistical analysis. Post hoc Tukey’s multiple comparisons test shows ** *p* < 0.01, **** *p* < 0.0001 versus control group; ## *p* < 0.01 versus insulin-alone group.

SH-SY5Y #9 cells were first differentiated with RA for 5 days and then cultured for 24 h under normal conditions or with high glucose (30 mM) plus high insulin (100 nM) to induce an insulin-resistant state. After such treatment, cells under both conditions were stimulated with 100 nM insulin w/o the presence of 100 nM orforglipron for 1 h. Cells then were collected and total protein was extracted for Western blot analysis. Although insulin stimulated Akt phosphorylation under both normal and insulin-resistant conditions, as shown in Figure 6A and compared to the normal condition, insulin-mediated Akt activation was significantly impaired during insulin resistance, with pAkt protein levels reduced by 56%. Nevertheless, when cells were treated with insulin + orforglipron (100 nM), pAkt protein levels were significantly elevated by approximately 140% of the insulin-alone levels in both conditions (Figure 6A). This indicates that orforglipron can augment neuronal pAkt signaling under both normal and insulin-resistant conditions. Subsequent experiments in differentiated #9 cells (Figure 6B) demonstrated that although orforglipron treatment alone did not significantly alter pAkt levels, it significantly amplified insulin-induced pAkt under both normal and insulin-resistant conditions (Figure 6B), showing its potential effect on neuroprotection by enhancing insulin signaling.

### 3.8. Orforglipron Brain Penetration

As a simple measure of orforglipron brain uptake, its concomitant concentrations in plasma and brain were quantified in male and female rats at 4 h following its systemic (i.p.) administration. This time was selected in light of the reported 4.49 h elimination half-life of orforglipron in rodents [50]. Plasma, brain, and CSF concentrations are shown in Table 1. Notably, there was no significant difference between concentrations measured in males vs. females across tissues. The brain/plasma and CSF/plasma ratios were 0.0078 (combining genders).

## 4. Discussion

Neurodegenerative disorders represent a leading worldwide cause of disability and mortality, impacting more than 57 million people annually [51]—a figure expected to double every 20 years. In the face of this growing disease burden, no cures are currently available, and treatment options remain wholly inadequate. They are limited by disease heterogeneity, an insufficient understanding of disease mechanisms, and a silent and protracted preclinical phase of disease development that makes early diagnosis and therapeutic intervention challenging. Insulin signaling, best known for its role in systemic glucose homeostasis, is a key regulator of synaptic plasticity, mitochondrial function, glial activity, and neuronal survival in healthy brain [52,53]. It functions as a crucial neuroprotective mediator that modulates the balance between cell survival and apoptosis, in large part via the PI3K/Akt pathway [54].

In brain insulin resistance, which is highly prevalent in neurodegenerative disorders [22,55], repressive serine phosphorylation of IRS-1 (S312/S616/S636) inhibits PI3K/Akt signaling, and this molecular roadblock thereby disrupts downstream insulin signaling. This culminates in impaired synaptic plasticity, glial activation and inflammation, compromised mitochondrial function, and declines in proteostasis mechanisms to clear misfolded and aberrant proteins such as α-synuclein and amyloid-β. These processes generate a deleterious positive feedback loop to further amplify impairments in insulin signaling, neurological function, and homeostasis. A treatment strategy with the potential to target multiple drivers of neurodegenerative disease pathology, including brain insulin resistance, neuroinflammation, cytotoxicity, and oxidative stress, may be more effective than existing single-pronged therapeutic options. GLP-1R stimulation activates similar pathways to those de-activated consequential to insulin resistance, and multiple preclinical studies and early human trials have reported broad GLP-1R-mediated neuroprotective, neurotrophic effects that, together, with insulin re-sensitization, underpin the diverse range of GLP-1R mediated pleotropic effects [9,22,56].

In the current study, we utilized a variety of human cellular models to evaluate the potential neuroprotective actions of the first small-molecular-weight GLP-1R medicine to gain clinical approval, orforglipron. These cellular models include two genetically modified SH-SY5Y neuronal cell lines: one overexpressing the human GLP-1R by 2-fold [42] (SH-SY5Y #9 in undifferentiated and differentiated states) and another expressing *Gaussia* luciferase specifically in the endoplasmic reticulum (SH-SY5Y^GLuc-ASARTDL^) [44]. Additionally, a human microglial cell line (HMC3), known to express the GLP-1R [25], was utilized to assess anti-inflammatory properties of orforglipron, expanding our evaluations of potential therapeutic effects on the CNS effects of orforglipron on glial cells. Notably, the selected cell lines are of human origin, as orforglipron is known to selectively activate the human GLP-1R but to not bind or activate the wild-type mouse/rat GLP-1R [57,58]. Our studies of neurotoxicity and neuroinflammation collectively demonstrate that orforglipron exerts multifaceted neurotrophic, neuroprotective, and anti-neuroinflammatory actions. This is the first report on the characterization of orforglipron in relation to potential actions of relevance to neurological disorders.

In addition to their high expression on pancreatic β-cells and presence within the cardiovascular system, gastrointestinal tract, kidney, and lung, GLP-1Rs are widely expressed throughout the brain—particularly in the hypothalamus, cerebral cortex, hippocampus, and brainstem, as well as within the limbic and reward-related circuits [59,60]. GLP-1R activation triggers the cAMP/PKA and PI3K/Akt pathways, in common with insulin receptor signaling. Similarly to peptide GLP-1RAs that our group has previously evaluated [25,42,61,62], orforglipron induces a time- and dose-dependent increase in intracellular cAMP levels in SH-SY5Y #9 neuronal cells (Figure 1). cAMP activity within neurons blocks downstream cellular apoptosis mechanisms [63]. This activity mirrors our observations in SHSY-5Y #9 cells of orforglipron’s neurotrophic and neuroprotective effects against glutamate challenges at concentrations as low as 1 nM (Figure 2). Additionally, orforglipron (at 10 nM and 100 nM) dose-dependently attenuated glutamate-induced endoplasmic reticulum (ER) stress (Figure 4). Excitotoxicity is a key driver of ER homeostasis disruption and is a hallmark of neurodegenerative disease and injuries [46,64,65]. Notably, orforglipron alone did not alter the baseline ER stress response, signifying a selectivity for pathological conditions without interfering with physiological ER function. Collectively, the data suggest that orforglipron has the ability to stabilize ER calcium and ER proteostasis.

Additionally, orforglipron reduced oxidative stress in neuronal cells challenged with an oxidative insult across a broad concentration range. It significantly lowered ROS levels across all evaluated states when assessed under normal culture conditions, but only mitigated ROS levels achieved by low oxidative stress under an insulin resistance setting (Figure 3). These results suggest that the anti-oxidative action of orforglipron is partly attenuated during insulin resistance. Using human microglial HMC3 cells, we demonstrated that orforglipron potently suppressed LPS- or glutamate-induced pro-inflammatory cytokine production (exemplified by IL-6, IL-8, and MCP-1) (Figure 5), which reveals that orforglipron is a potential modulator of microglial reactivity eliciting anti-neuroinflammation effects. Aligning with this, examining key signaling protein pAkt levels in neuronal cells, orforglipron enhanced Akt phosphorylation in response to insulin stimulation under both normal and insulin-resistance conditions (Figure 6). In both conditions, our data support orforglipron as a promising enhancer of neuronal Akt signaling, highlighting synergistic potential with insulin pathways.

From a mechanistic perspective, orforglipron fundamentally differs in its GLP-1R interaction, as compared to peptide GLP-1 medicines. Notably, GLP-1R peptide agonists, which are similar to endogenous GLP-1, bind within the receptor’s extracellular orthosteric binding pocket. In contrast, orforglipron functions as an allosteric agonist [2] by binding within a separate transmembrane pocket. Drug engagement generates an active receptor conformation that effectively couples to Gs proteins to trigger a Gs-biased signaling profile and induce intracellular cAMP generation [58]. In our prior studies of peptide-based GLP-1R agonists (reviewed in [17,18,19,20,21,22]), we validated our cellular studies in immortal human cell lines with parallel ones in primary neuronal cultures as well as in rodent disease models. This is more difficult to undertake with orforglipron in the light of its specificity to the human GLP-1R [2], which is a limitation of the current study.

Whereas orforglipron appears to possess promising actions in neuronal and glial cell culture models predictive of neuroprotective, neurotrophic, and anti-neuroinflammatory activity, blood–brain barrier permeability is required for a drug to be effective within the CNS. The brain uptake of most peptide-based therapeutics is, in general, far lower than small-molecular-weight drugs [66,67]. In this regard, two recent studies have evaluated the acute brain uptake of a range of clinically approved peptide-based GLP-1R agonists in mice [68,69]. Brain parenchymal levels following systemic drug administration were low but nevertheless quantifiable for some drugs, with brain entry rates varying across 15-fold between agents, whereas for other GLP-1 peptide-based medicines, particularly acylated and PEGylated ones, brain levels were below detectable limits [68,69]. Data on brain uptake in humans is largely lacking, but the GLP-1R peptide agonist exenatide has a reported CSF/plasma concentration ratio of 1–2% [70,71], and has demonstrated both positive actions and a lack of action in human neurodegenerative disorders [70,72,73,74,75,76,77].

As an initial estimate of the CNS penetration potential of orforglipron, we calculated its standard Central Nervous System Multiparameter Optimization (CNS MPO) desirability score [78], which equaled 1.8. This MPO score is relatively low, and notably below the commonly preferred threshold of ≥4 for higher-probability CNS penetration of marketed drugs. Additionally, orforglipron does not align with the Lipinski CNS “Rule of 5” score [78]. On quantifying concurrent brain and plasma drug levels achieved in rats following a clinically translatable systemic orforglipron dose, the brain/plasma ratio was similar across males and females, and equal to 0.0078 across genders, as was the CSF/plasma drug ratio. Specifically, brain levels achieved only 0.78% of simultaneous plasma levels. This low brain uptake is less than expected from orforglipron’s MPO score, but aligns with reports that it is both highly plasma-protein-bound (>99%) and, notably, a substrate for the p-glycoprotein transporter [79]. This cell membrane efflux pump, in addition to its presence on the apical membrane of intestinal, renal, and hepatic cells, is localized at the blood–brain barrier, where it reduces the brain uptake of its substrates [80]. Nevertheless, it is possible that even with a low brain uptake, small-molecule orforglipron may have an advantage over available peptide-based GLP-1R agonists in potentially providing meaningful and beneficial CNS-mediated actions. In this light, the promising neurotrophic, neuroprotective, and anti-inflammatory actions of orforglipron in neuronal and glial cell culture models in the present study support further studies to specifically evaluate potentially beneficial drug actions beyond orforglipron’s T2DM and weight loss ones.

## Figures and Tables

**Table 1:** Orforglipron plasma, brain, and CSF levels in rat following systemic dosing. Orforglipron (3.6 mg/kg, i.p.) was administered, and plasma, brain, and CSF were sampled 4 h later. Drug concentrations were then evaluated by UHPLC-MS to determine brain/plasma and CSF/plasma ratios.

	Plasma (pmol/mL)	Brain (pmol/g)	CSF (pmol/mL)	Brain/Plasma Ratio	CSF/Plasma Ratio
Male	809.04 ± 38.3 (n = 4)	5.80 ± 0.78 (n = 4)	4.57 ± 1.98M (n = 3)	0.0071	0.0058
Female	663.19 ± 49.45 (n = 4)	5.45 ± 0.44 (n = 4)	8.08 ± 6.59 (n = 2)	0.0084	0.0109
Combined	736.1 ± 40.0 (n = 8)	5.62 ± 0.62 (n = 8)	5.97 ± 2.50 (n = 5)	0.0078	0.0078

## Data Availability

Data is available at reasonable request of the authors.

## Supplementary Materials

The following supporting information can be downloaded at: https://www.mdpi.com/article/10.3390/cells15141301/s1.
