# Supplementary material for: Orforglipron, a Small-Molecule Glucagon-like Peptide-1 Receptor Agonist (GLP-1RA), Has Neuroprotective and Anti-Inflammatory Effects
Source: Cells. 2026 Jul 21;15(14):1301. doi: 10.3390/cells15141301 (PMC13406627; doi:10.3390/cells15141301)

Supplemental Figure S1

A 48 h exposure to orforglipron reduces proinflammatory cytokine levels in human microglial cells (HMC3) challenged with glutamate. HMC3 cells were challenged with (glutamate (25 or 50 mM) w/o the presence of orforglipron (100 nM) for 48 h. Proinflammatory protein (IL-6, and MCP-1) levels were then quantified in culture media by ELISA (n = 4–6). One-way ANOVA was used for statistical analysis. Post hoc Tukey's multiple comparisons test shows * $p < 0.05$, ** $p < 0.001$, **** $p < 0.0001$ versus glutamate comparison group.

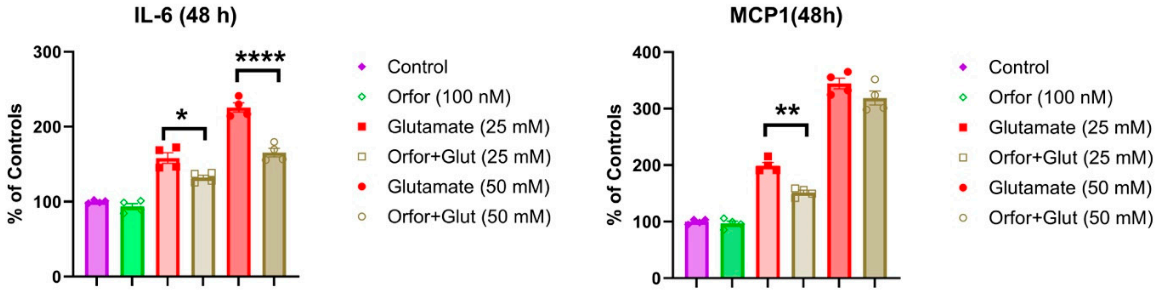

Supplement: Supplementary file 1 [file cells-15-01301-s001.zip › cells-4373453-supplementary.pdf]
